# Harnessing Machine Learning To Unravel Protein Degradation in Escherichia coli

**DOI:** 10.1128/mSystems.01296-20

**Published:** 2021-02-02

**Authors:** Natan Nagar, Noa Ecker, Gil Loewenthal, Oren Avram, Daniella Ben-Meir, Dvora Biran, Eliora Ron, Tal Pupko

**Affiliations:** a Shmunis School for Biomedicine and Cancer Research, George S. Wise Faculty of Life Sciences, Tel Aviv University, Tel Aviv, Israel; NYU School of Medicine

**Keywords:** protein degradation, proteomics, machine learning, SILAC

## Abstract

Degradation of intracellular proteins in Gram-negative bacteria regulates various cellular processes and serves as a quality control mechanism by eliminating damaged proteins. To understand what causes the proteolytic machinery of the cell to degrade some proteins while sparing others, we employed a quantitative pulsed-SILAC (stable isotope labeling with amino acids in cell culture) method followed by mass spectrometry analysis to determine the half-lives for the proteome of exponentially growing Escherichia coli, under standard conditions. We developed a likelihood-based statistical test to find actively degraded proteins and identified dozens of fast-degrading novel proteins. Finally, we used structural, physicochemical, and protein-protein interaction network descriptors to train a machine learning classifier to discriminate fast-degrading proteins from the rest of the proteome, achieving an area under the receiver operating characteristic curve (AUC) of 0.72.

**IMPORTANCE** Bacteria use protein degradation to control proliferation, dispose of misfolded proteins, and adapt to physiological and environmental shifts, but the factors that dictate which proteins are prone to degradation are mostly unknown. In this study, we have used a combined computational-experimental approach to explore protein degradation in E. coli. We discovered that the proteome of E. coli is composed of three protein populations that are distinct in terms of stability and functionality, and we show that fast-degrading proteins can be identified using a combination of various protein properties. Our findings expand the understanding of protein degradation in bacteria and have implications for protein engineering. Moreover, as rapidly degraded proteins may play an important role in pathogenesis, our findings may help to identify new potential antibacterial drug targets.

## Abstract (precis)

Bacteria use protein degradation to control proliferation, dispose of misfolded proteins, and adapt to physiological and environmental shifts, but the factors that dictate which proteins are prone to degradation are mostly unknown. In this study, we have used a combined computational-experimental approach to explore protein degradation in E. coli.

## INTRODUCTION

The degradation of intracellular proteins is a fundamental process of life and serves various important physiological functions, including removal of abnormal proteins and regulation of basic cellular processes (1–7). In eukaryotes, the covalent binding of a small protein, ubiquitin, marks proteins for degradation by the proteasome (8). In bacteria, ATP-dependent AAA+ (ATPases associated with cellular activities) proteases use ATP hydrolysis to fuel substrate degradation (7). Degradation of intracellular proteins in Gram-negative bacteria is mainly performed by five ATP-dependent AAA+ proteases: ClpAP, ClpXP, Lon, HslUV, and FtsH (5, 7, 9).

Since protein degradation is an irreversible process with a considerable damaging potential (10), protease activity has to be carefully regulated. Many factors were suggested for regulating degradation, mainly for eukaryotic cells. These include physical properties such as protein mass, isoelectric point, surface accessibility, structural disorder, and low-complexity regions (11–14), as well as sequence-related properties such as the N-end rule, PEST (sequence that is rich in proline, glutamic acid, serine, and threonine), destruction box, KEN box, and other sequence motifs (15–21). Sequence motifs that are involved in the regulation of protein degradation are known as “degrons.” It is assumed that these sequences are located at the C and N termini of proteolytic substrates (15, 19, 22–24). For example, it was suggested that ClpXP recognizes proteolytic substrates through five degron classes; three are located at the N termini of proteins (polar-T/ϕ-ϕ-basic-ϕ, NH_2_-Met-basic-ϕ-ϕ-ϕ-X5-ϕ, and ϕ-X-polar-X-polar-X-basic-polar, where ϕ represents hydrophobic amino acids and X any amino acid), and two are located at the C termini (LAA-COOH and RRKKAI-COOH). A proteolytic substrate can bear either a C-terminal motif, an N-terminal motif, or both (19). The LAA-COOH motif is similar to the SsrA tag (AANDENYALAA), which is known to be appended to the C termini of proteins for which translation cannot be completed (22), thereby targeting the tagged, defective protein to degradation by the ClpXP protease (19, 25). Several attempts have been made to systematically estimate the collective and/or individual contribution of known degradation-regulating factors. This was done either in the context of the overall variability observed for protein stability in bacteria (15) or in the context of the substrate repertoires of specific proteases (19, 20, 26, 27).

Over the past decade, it has become possible to track protein degradation *in vivo* at the global level, i.e., degradation profiles (28, 29). These profiles were determined by the heavy-light amino acid pulsed-SILAC (stable isotope labeling with amino acids in cell culture) technology followed by quantitative mass spectrometry (MS) (30) as well as other MS-based methods, for various organisms and in different physiological contexts (19, 27, 31–37). In the pulsed-SILAC setting, the isotopic ratios of the different mass labels, which are frequently used for differential expression analysis, are instead used to determine the dynamics of protein degradation (38). We used pulsed SILAC to determine protein half-lives in exponentially growing Escherichia coli. We then used statistical modeling of protein stability to classify each protein to one of three mutually exclusive stability groups that we termed as stable, slow-degrading, and fast-degrading proteins. We next searched for various features that characterize each of these stability groups and used them to train a machine learning classifier.

Machine learning approaches have proved useful for predicting various aspects of protein functions, including prediction of novel effector proteins in pathogenic bacteria, prediction of phosphorylation sites, and prediction of subcellular locations, to name but a few (39–45). A critical requirement for such a machine learning approach is reliable training data, which in the context of protein degradation are accurately determined protein half-lives. To this end, we used our data to develop machine learning classification algorithms to assign each cellular protein to one of the stability groups, based on its associated features, and reached an area under the receiver operating characteristic (ROC) curve (AUC) of 0.72.

## RESULTS

### Quantification of protein half-lives in E. coli.

**FIG 1:**
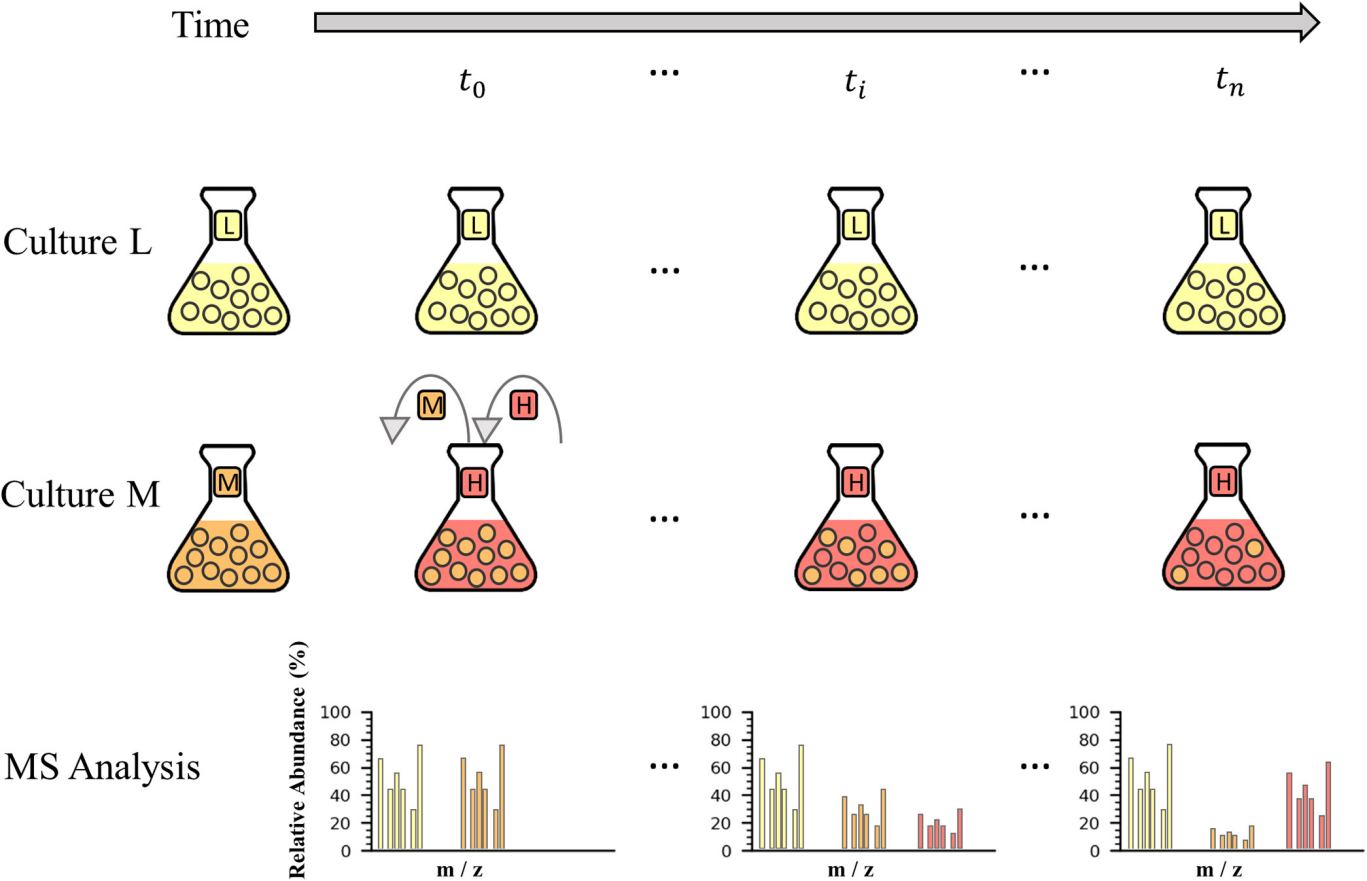
Pulsed-SILAC method illustration. E. coli cells were cultured in different SILAC media (culture L and culture M) containing either light (yellow) or medium (orange) lysine until full incorporation of the relevant isotope (leftmost Erlenmeyer flask in each culture). The gray arrow at the top represents the experiment timeline (during bacterial exponential growth phase). At *t*_0_, the medium lysine isotope of culture M is replaced by the heavy lysine isotope (red). Next, at each time point *t*_i_ (including *t*_0_ and *t*_n_), equal amounts of cells were sampled from culture L and culture M, mixed, and analyzed by mass spectrometry (MS). The resulting ratios of M/L isotopes over time measures the rate of protein degradation.

We measured protein half-lives in exponentially growing E. coli cells by applying pulsed SILAC to dividing cells followed by quantitative MS analysis of whole-cell extracts as a function of time (see Materials and Methods). This is an adaptation to bacteria of the experimental design described in reference 28. Briefly, the cultures were grown and passaged in the presence of either light (L) or medium (M) lysine isotopes until full incorporation of the label. When the cultures reached mid-exponential phase, the medium of the culture growing with the M lysine was replaced with medium containing the heavier (H) lysine isotope. The rate of protein degradation was inferred from the decreasing ratio of M/L isotopes over time (Fig. 1). In total, we identified and quantified 1,602 proteins (see Data Set S1 in the supplemental material). This value is within the range that was reported for other SILAC experiments in bacteria, although with only a double labeling, not triple (34, 46). Out of this subset, we estimated the half-life of 1,149 proteins (Data Set S2; see Materials and Methods for filtering criteria).

10.1128/mSystems.01296-20.1 DATA SET S1 A total of 1,602 proteins that were quantified using pulsed-SILAC followed by LC-MS analysis. Download Data Set S1, XLSX file, 1.1 MB. Copyright © 2021 Nagar et al. 2021 Nagar et al. This content is distributed under the terms of the Creative Commons Attribution 4.0 International license.

10.1128/mSystems.01296-20.2 DATA SET S2 Data for classification of 1,149 E. coli proteins to fast-degrading, slow-degrading, or stable (the last column is the protein half-life and is included for clarification purposes only). The “label” column describes protein stability, where 0, 1, and 2 represent slow-degrading, fast-degrading, and stable proteins, respectively. The proteins are indicated by their protein identifiers and gene names. Download Data Set S2, XLSX file, 2.1 MB. Copyright © 2021 Nagar et al. 2021 Nagar et al. This content is distributed under the terms of the Creative Commons Attribution 4.0 International license.

### Statistical modeling of protein stability reveals that only a small subset of proteins undergoes rapid degradation.

The half-life values of the proteins vary dramatically, ranging from minutes to a few days. We classified the quantified proteins to either a stable or degradable group by selecting one of two nested models of protein degradation (see Materials and Methods). The first model states that for a given protein, the exponential decrease in M/L ratio over time is governed solely by protein dilution due to cell division, whereas the second model states that this decrease results from the combined effects of protein dilution and degradation. A total of 408 proteins for which the dilution model was significantly less likely than the degradation and dilution model were termed degradable, whereas the other 741 proteins were termed stable (S). This distribution indicates that for the majority of E. coli proteins expressed under standard conditions, degradation is undetectable. While most proteins are not degraded under standard conditions, we observed a fraction of unstable proteins that agrees with the 2 to 7% (out of the total protein content) unstable proteins predicted from previous experiments (47–49).

Among the fast-degrading proteins, we identified several proteins previously reported to have short half-life values, such as RNA polymerase sigma factor (RpoS) and DNA protection during starvation protein (Dps), with half-lives of approximately 2 and 10 min during the exponential phase, respectively (50, 51). In this study, they were classified as fast-degrading proteins with half-life values of 5.8 and 6.5 min. An extensive literature survey revealed that out of 72 proteins identified by us as fast-degrading proteins (see below), 21 were previously reported as being prone to degradation, while the remaining 51 are newly identified as fast-degrading proteins (Table S1).

10.1128/mSystems.01296-20.6 TABLE S1 Novel as well as previously reported fast-degrading proteins identified in this study. Download Table S1, PDF file, 0.1 MB. Copyright © 2021 Nagar et al. 2021 Nagar et al. This content is distributed under the terms of the Creative Commons Attribution 4.0 International license.

**FIG 2:**
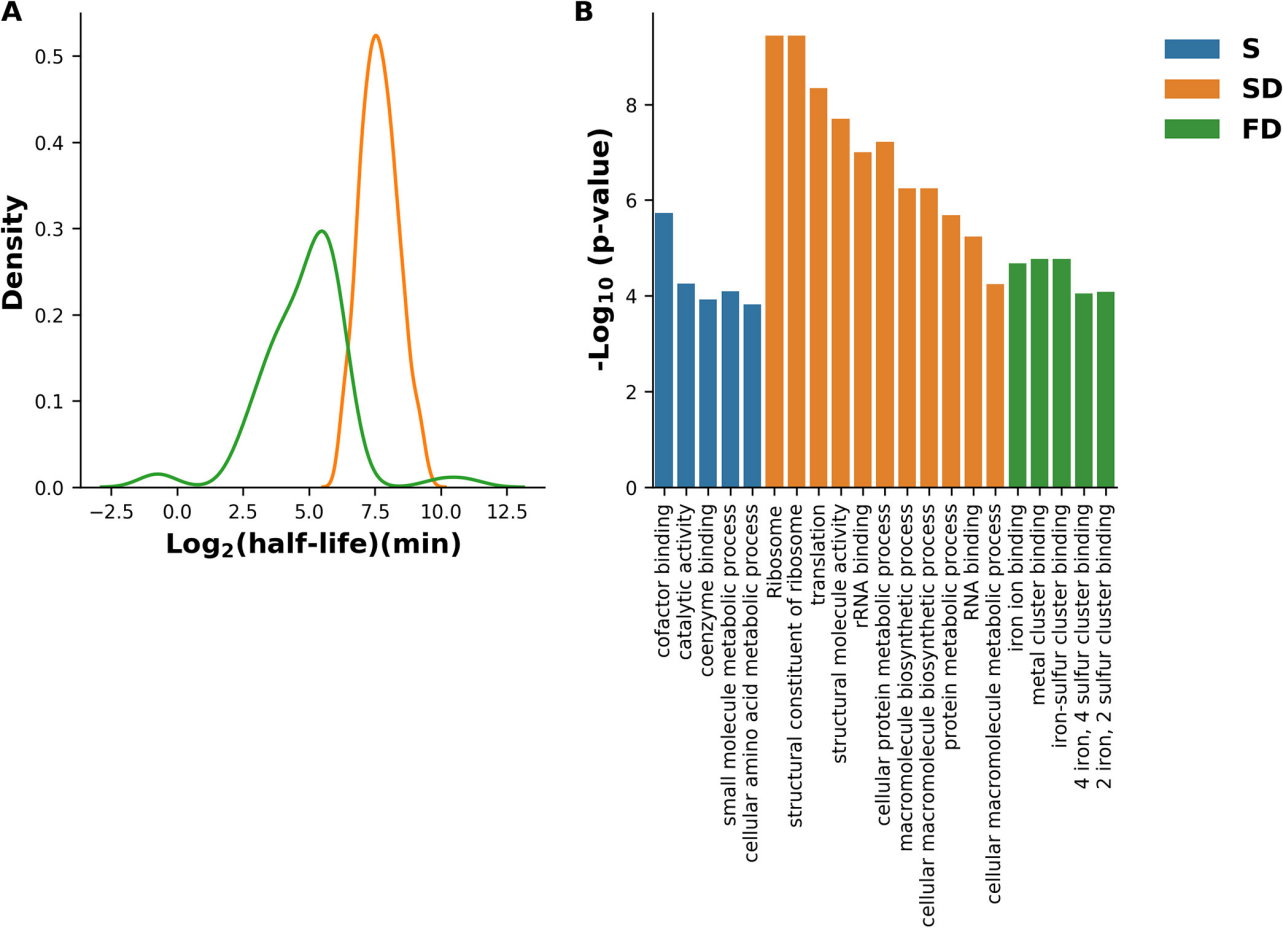
Determination of protein half-lives. (A) The distribution of the half-lives of 408 degradable E. coli proteins is composed from two distinct subpopulations of slow-degrading (SD) (*n* = 335) and fast-degrading (FD) (*n* = 74) proteins. The bins are log_2_ increments. (B) Enrichment analysis demonstrates functional differences between fast-degrading and slow-degrading/stable proteins based on GO annotations of molecular functions and biological processes as well as KEGG pathway annotations.

It was previously suggested that the degradable fraction of the proteome of E. coli is composed of rapidly and slowly decaying components (47, 48). The alternative hypothesis is that there exists a single component with high variance. We used an expectation maximization algorithm to estimate the maximum likelihood of the two-component mixture model and compared it against a single-component model (see Materials and Methods). The expectation maximization algorithm identified two distinct distributions, ∼Norm(7.64, 0.72) and ∼Norm(5.58, 2), with a log likelihood of −669.5. Likelihood ratio testing by parametric bootstrapping between one-component and two-component-mixture models confirmed the latter (*P* value < 0.001), indicating that the degradable group is most likely composed of two distinct protein subpopulations. The expectation maximization algorithm also assigns probabilities for being a member of a specific distribution. By applying a probability threshold of 0.5, we obtained 334 and 74 proteins that are distributed according to ∼Norm(7.64, 0.72) and ∼Norm(5.58, 2) and are therefore termed slow and fast degrading, respectively (Fig. 2A). The proteins YgcE and HolC, which were assigned by the expectation maximization algorithm to the fast-degrading group, had much longer half-lives than proteins in the slow-degrading group (more than 16 h). We suspect that these two proteins, which constitute the right-tail density of the half-life distribution of the fast-degrading proteins, were attributed to this group because the extremity of their half-lives is significantly inconsistent with the narrow distribution of half-lives of the slow-degrading proteins. We therefore decided to include YgcE and HolC in the group of stable proteins, and thus, 72 proteins were classified as fast-degrading proteins.

Since the culture was sampled several times during exponential growth, we hypothesized that most of the stable and slow-degrading proteins would be directly involved in growth. It seems unlikely that proteins that are indispensable for growth would be targeted for degradation under conditions in which they are needed most. To test this hypothesis, we analyzed the enrichment of gene ontology (GO) molecular function and biological process annotations of stable, slow-degrading, and fast-degrading proteins. Slow-degrading and stable proteins were found to be mostly enriched for annotations related to metabolism, biosynthesis, and growth, including catalytic activity, cofactor and coenzyme binding, and translation. In contrast, fast-degrading proteins were found to be enriched for annotations related to metal binding (Fig. 2B). We suspect that this result reflects the lack of trace metals in the growth medium, suggesting that some metal-binding proteins are rapidly degraded in the absence of metals. Such proteins were previously shown to be degraded by AAA+ ATP-dependent proteases (52). Conversely, other metal-binding proteins, such as the zinc binding protein GlyA, the copper binding protein CopA, and the manganese binding protein PepA, are members of the stable protein group. These and other proteins are also defined as cofactor binding proteins and were found to be enriched in the stable protein group (Fig. 2B). To better understand the biological roles of fast-degrading proteins, we also analyzed the annotations that were not significantly enriched. Several fast-degrading proteins were found to be either poorly characterized or involved in various processes, including response to diverse stress conditions, including cold, oxidative stress, and DNA damage, as well as in proteolysis, regulation of transcription, and biofilm formation (Table S1). The number of identified peptides for the degraded proteins is given in Data Set S3.

10.1128/mSystems.01296-20.3 DATA SET S3 Number of peptides used to identify the 1,149 proteins for which the half-life was determined. Download Data Set S3, XLSX file, 0.05 MB. Copyright © 2021 Nagar et al. 2021 Nagar et al. This content is distributed under the terms of the Creative Commons Attribution 4.0 International license.

### Statistical comparison between fast-degrading, slow-degrading, and stable proteins.

**FIG 3:**
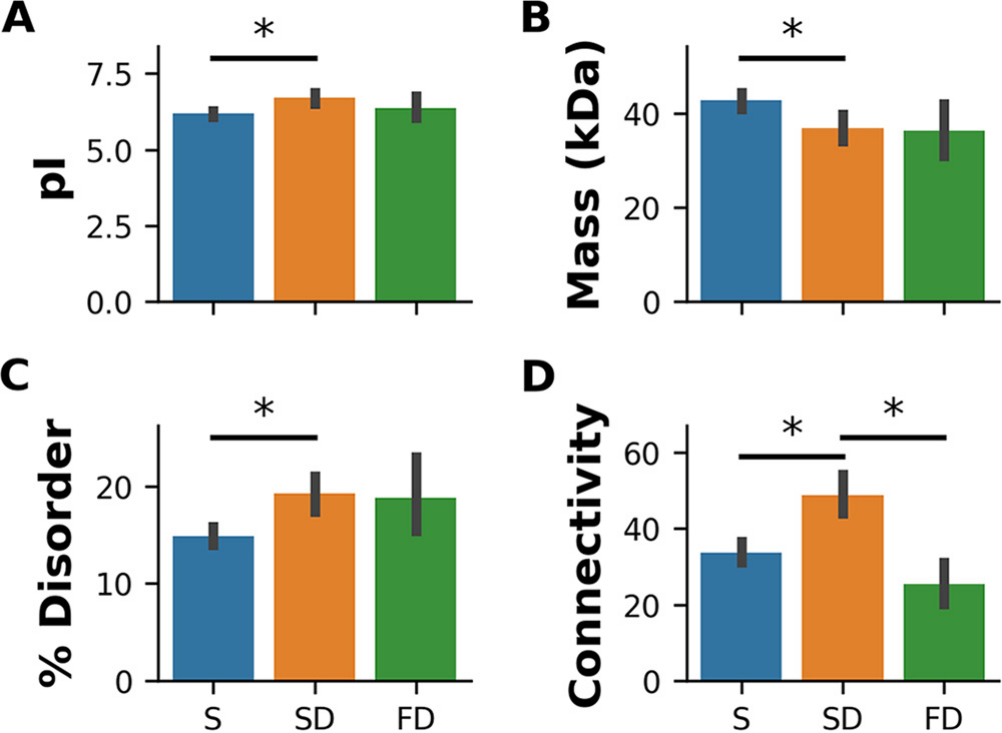
Comparison of protein properties. (A) Isoelectric point; (B) molecular mass; (C) predicted percentage of disordered amino acids; (D) connectivity. The three stability groups are stable (S), slow degrading (SD), and fast degrading (FD) proteins. *, *P* value < 0.005, one-way ANOVA followed by Tukey’s test. Error bars indicate 95% confidence intervals of the means for each property in each stability group.

Previous studies have reported that structural, physical, and sequence properties, as well as protein-protein interaction network (PPIN)-associated features, correlate with protein degradation (11, 12, 14, 19, 53, 54). To test if the fast-degrading, slow-degrading, and stable proteins differ in such properties, we conducted a comparative analysis of various protein-related features across the three protein stability groups. We first analyzed the physicochemical, structural, and PPIN properties of the three groups. Stable proteins were found to be slightly more acidic and larger than slow-degrading ones (Fig. 3A and B). However, no significant difference was found between the isoelectric points and masses of fast-degrading and slow-degrading proteins or of fast-degrading and stable proteins. This suggests that the degradation of fast-degrading proteins is governed by factors other than simple physical properties. Interestingly, stable proteins were found to be significantly less disordered than fast- and slow-degrading proteins (Fig. 3C). Slow-degrading proteins were found to be significantly more connected in the PPIN than fast-degrading and stable ones (Fig. 3D), suggesting that slow-degrading proteins interact with a larger number of proteins, either physically, functionally, or both.

We next analyzed several sequence properties of the three stability groups. The recognition of proteolytic substrates in bacteria is thought to be mediated by short sequence motifs, termed degrons, which are present at the terminal regions of the substrate. Properties that directly or indirectly capture this information are therefore expected to be highly predictive of protein degradation in bacteria. The N-end rule (53) and several other C- and N-terminal motifs that were previously reported as important in protein degradation were collected. The frequency of each amino acid at the second position of the N terminus (after the formylaminoacylated formylatable methionine [fMet]) was used to capture the N-end rule (see Materials and Methods). In addition, the number of occurrences of each amino acid grouped into five physicochemical properties at the second position of the N terminus was also used to capture the N-end rule. Besides the N-end rule, the numbers of occurrences of few N- and C-terminal sequence motifs that are thought to be recognized by the ClpXP protease were also analyzed (19). Together, the N-end rule and ClpXP recognition signals constitute the most established determinants of protein degradation in bacteria. Interestingly, no significant dependency was found between any of these features and protein stability (Fig. S1), suggesting that these signals may promote degradation of a small fraction of bacterial proteins.

10.1128/mSystems.01296-20.4 FIG S1 Normalized frequency of previously reported characteristics of unstable proteins in bacteria. The N-end rule (A. Varshavsky, Genes to Cells 2:13–28, 1997, https://doi.org/10.1046/j.1365-2443.1997.1020301.x), three N-terminal (NM1 to NM3), and two C-terminal (CM1 and CM2) recognition sequences of ClpXP (J. M. Flynn, S. B. Neher, Y. I. Kim, R. T. Sauer, and T. A. Baker, Mol Cell 11:671–683, 2003, https://doi.org/10.1016/S1097-2765(03)00060-1) are shown. The number of occurrences of each of the 20 amino acids, represented by single letters (ACDEFGHIKLMNPQRSTVY) and the number of occurrences of aliphatic (IVL), aromatic (FYWH), charged (KRDE), tiny (GACS), or diverse (TMQNP) amino acids at the second position of the N terminus summarize the N-end rule. The amino acid tryptophan (W) was not observed at this position, and the CM2 motif was not found, in any of the proteins. Protein stability was not found to be dependent on any of these sequence motifs (chi-square test followed by Benjamini-Hochberg FDR correction). Stability groups include stable (S), slow-degrading (SD), and fast-degrading (FD) proteins. The presence or absence of a sequence motif is designated by a plus or minus sign, respectively. Download FIG S1, PDF file, 0.2 MB. Copyright © 2021 Nagar et al. 2021 Nagar et al. This content is distributed under the terms of the Creative Commons Attribution 4.0 International license.

### Machine learning to predict fast-degrading proteins.

**FIG 4:**
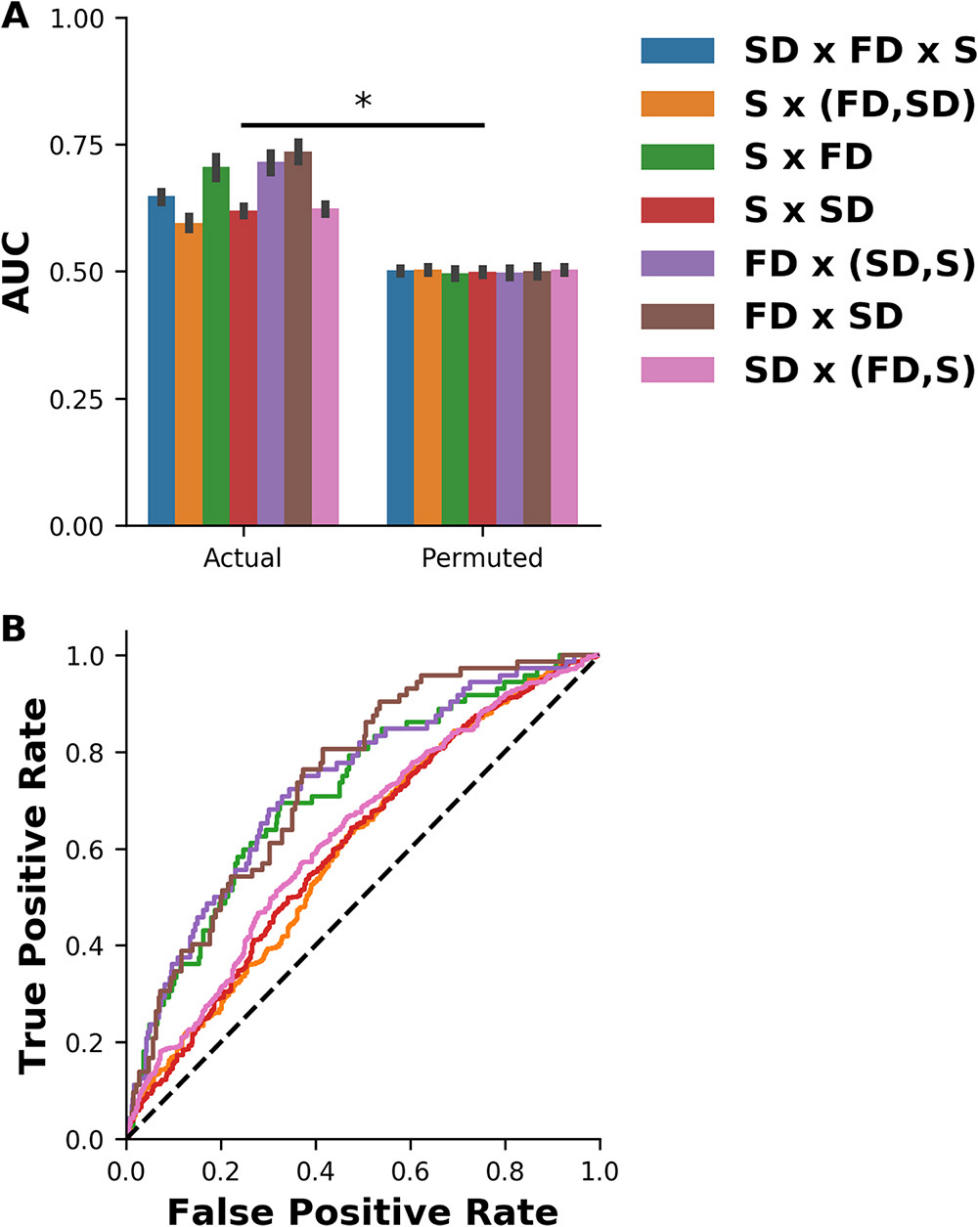
PPIN and physical protein features discriminate fast-degrading proteins from stable/slow-degrading ones. (A) Classification of proteins to S, SD, and FD proteins using logistic regression trained with 188 physicochemical, structural, and PPIN-related features is significantly better than random. Stability groups (S, SD, and FD) are in parentheses when one stability group was compared against the rest of the groups. All models trained with the actual data were compared using paired *t* test to their corresponding permuted data set. *, *P* value < 0.0001, paired *t* test followed by FDR correction. The performances obtained using the actual data sets were significantly higher than their corresponding permuted data sets. The AUC of the actual data was estimated by 10 repeats of 10-fold cross-validation while the AUC corresponding to permuted data is an average of 100 repeats of 10-fold cross-validation, where each repeat is a different permutation of the class labels. For each comparison, the error bars indicate 95% confidence intervals of the mean AUC across all 10-fold cross-validation runs. (B) ROC AUC curves for all classification setups excluding the multiclass classification FD × SD × S. For each comparison, the curve was constructed based on a single, representative 10-fold cross-validation run.

We observed small yet statistically significant differences in the percentage of structural disorder, mass, isoelectric point, and node connectivity among the various stability groups. We next tested whether these and other presumably informative features could be used to predict the stability category of each protein. To this end, we applied machine learning classification algorithms to find a function between the set of features and the stability group, i.e., to train a machine learning classifier. An accurate classifier would predict the correct label for “unseen” data. To test the accuracy, a part (fold) of the data is treated as unknown while the remaining folds are used to train the classifier (see Materials and Methods). We included all features that are potentially related to protein stability, including physicochemical, structural, sequence, and PPIN-related features, as well as features that integrate the node connectivity of each protein with its structural and physicochemical attributes. Overall, 188 features were collected for the classification (Data Set S2). The performance of the classifier is measured in terms of AUC, where an AUC of 1 indicates perfect classification and an AUC of 0.5 corresponds to a random classification. The highest accuracies were obtained when fast-degrading proteins were not grouped with either slow-degrading or stable ones. The highest score (AUC, 0.74 ± 0.01) was obtained when comparing fast- versus slow-degrading proteins (Fig. 4). All these comparisons are significantly better than a classifier trained on permuted data sets (all *P* values < 0.001), confirming that the feature set used for training the classifier contains features that are significantly correlated with protein degradation. The quality of discrimination between the fast-degrading and the slow-degrading and stable proteins is of special interest, because good discrimination will enable the computational prediction of fast-degrading proteins. In this setting, our classifier achieved an AUC of 0.72 ± 0.01, suggesting that intrinsic protein properties as well as PPIN-related features are predictive of protein stability in E. coli. Interestingly, the most informative features across all the comparisons were PPIN-related features, suggesting that fast-degrading proteins share similar network properties (Fig. S2). Of note, including GO annotations as features did not improve the accuracy of the classifications (data not shown).

10.1128/mSystems.01296-20.5 FIG S2 Top five features associated with each discrimination. Stability groups (S, SD, and FD) are in parentheses when one stability group was compared against the rest of the groups. Con, node connectivity in the PPIN. The node2vec features are designated with the prefix “V”. Download FIG S2, TIF file, 0.6 MB. Copyright © 2021 Nagar et al. 2021 Nagar et al. This content is distributed under the terms of the Creative Commons Attribution 4.0 International license.

## DISCUSSION

The degradation of intracellular proteins is important for the regulation of cellular processes and serves as a mechanism for protein quality control. Hence, the quantification of protein half-lives and the elucidation of factors determining degradation dynamics are critical for the understanding of protein activity regulation. In this work, pulsed SILAC followed by liquid chromatography-mass spectrometry (LC-MS) was applied to explore protein degradation in E. coli during the exponential phase of growth. This enabled monitoring of the degradation of proteins that were present in the cell at the early exponential phase to mid-exponential phase of growth, at the proteome level. A key step for understanding protein degradation is the reliable quantification of the half-lives of all proteins expressed under a given condition. To achieve this, we determined and modeled the degradation of 1,149 proteins, which constitute nearly half of the expressed proteins in E. coli (55), providing the largest data set of its kind for protein half-lives for this species. The use of the log-likelihood ratio test combined with the expectation maximization algorithm to choose the most likely mode of degradation for each protein revealed three distinct stability groups: stable, slow-degrading, and fast-degrading proteins. The vast majority of the proteins were classified as highly stable or slow degrading (66% and 29.1%, respectively). The remaining 6.3% were found to be fast degrading, with half-lives ranging from 70 min to less than a minute (Fig. 2A). These values are in agreement with an early study that found that only 2 to 7% of the E. coli protein content undergoes rapid degradation during the exponential growth phase (47). We assume that most of the proteins that were not identified in this study either are not expressed or are too unstable to be detected in our experimental setting. In this context, we encountered what seems to be a typical limitation of pulsed-SILAC methods (28, 34), in which respective peptides are undetectable for certain proteins at some of the sampled time points, leading to some loss of information.

A similar pulsed-SILAC approach was previously taken to track protein degradation at the transition from exponential to stationary phase of growth in Staphylococcus aureus (34). In this setting, most proteins that undergo rapid degradation are proteins that are essential in substantial amounts during the exponential phase, such as ribosomal proteins and anabolic or catabolic enzymes. In our experimental setting, proteins required for growth were found to be mostly stable or slow degrading, while the fast-degrading proteins had diverse roles, including metal binding, response to various stresses, and transcriptional regulation (Fig. 2B and Table S1). This suggests that protein degradation is differentially regulated at the various stages of growth and that proteins that are unstable during growth may become stable under stress or starvation, and vice versa. Indeed, additional experiments are needed to test the effects of more specialized conditions, such as various stresses, alternative nutrient conditions, or the presence of trace metals, on protein half-lives *in vivo*.

The fast-degrading proteins have a high turnover during growth. What may be the biological significance of such a phenomenon, i.e., why should evolution favor a state in which proteins are continuously transcribed and translated only to immediately be degraded? We propose six possible explanations: (i) These proteins harbor degrons recognized by the AAA+ ATP-dependent proteases which could not be eliminated in the course of evolution due to structural or functional constraints. (ii) Rapid accumulation of fast-degrading proteins can be achieved by stopping their degradation, e.g., by the inhibition of specific proteases or modulation of adaptors. Thus, the degradation of such proteins is used as a regulatory switch that keeps their concentration low at exponential phase yet allows a rapid increase in their concentration upon an environmental change (56, 57). (iii) Proteins that are involved in specific steps of the cell cycle might oscillate between cycles, which may cause us to identify them as fast-degrading proteins (58, 59). (iv) Protein degradation adjusts the level of proteins which are members in heterocomplexes and are synthesized at different levels. (v) These proteins are prone to misfolding under exponential growth conditions, and most of the proteolysis is of the misfolded variants. (vi) The instability of these proteins is protease independent. Clearly, the current data do not allow us to determine the relative contribution of each of these possible factors.

Proteolysis was previously suggested to have a role in regulating the activity of RpoS and Dps proteins. RpoS regulates gene cascades that are involved in response to various stress conditions, including oxidative stress, extreme temperature, pH, and osmolarity as well as DNA damage. The Dps protein binds and thereby protects DNA from oxidative stress. It was suggested that inhibition of their constant degradation by AAA+ ATP-dependent proteases during the exponential phase is important for their rapid accumulation following stress, which, in turn, enables them to respond quickly to the stress signal (60). We note that testing the biological effect of protein stability and the role of specific residues in governing protein stability, *in vivo*, is a challenging task since residues may play multiple roles, e.g., in protein folding, interaction with other molecules, and stability (50).

Studying protease-independent stability can be done by systematic determination of the stability of purified proteins *in vitro*. Another possibility is to study degradation rates *in vivo*, in which all AAA+ ATP-dependent proteases are knocked out. In the case of the essential FtsH protease (61), such studies can be conducted with conditional mutants for this gene (62). The effects of various physical (temperature and osmolarity) and biological (medium composition and introduction of stress) factors on protein stability remain to be studied. Moreover, the effect of ATP-independent proteases remains to be discovered. Finally, it is of interest to discover if, and how, bacteriophages manipulate protein degradation rates to their benefit.

Once we obtained reliable information on protein degradation, we could focus on the more challenging problem of identifying key differences between fast-degrading proteins and the rest of the quantified proteome and using them for prediction. A prerequisite for this challenge is to objectively sort the proteins (in the training set) into different stability groups. In this study, we employed likelihood ratio tests together with expectation maximization, thereby avoiding arbitrary cutoffs for discriminating between the stability groups. This objective criterion revealed the existence of three distinct stability groups. We collected several features previously reported as correlated with degradation, as well as other potentially predictive ones. We showed that physicochemical and PPIN properties are more correlated with degradation than previously described degrons (Fig. S1 and S3). This implies that both substrate specificity and substrate selectivity of AAA+ ATP-dependent proteases are broader than previously thought. Our machine learning algorithm combines both structural and physicochemical features with PPIN-related features to classify proteins to different stability groups (Fig. 4). As the degradation of some bacterial proteins was suggested to be of clinical relevance (63–65), our results may lead the foundations for the discovery of novel drug targets. In this context, it would be interesting to estimate how well our machine learning approach generalizes to evolutionarily related proteobacteria and diverged bacterial species, including pathogenic strains.

## MATERIALS AND METHODS

### Reagents and bacteria.

MgSO_4_, NaCl, NH_4_Cl, CaCl_2_, glucose, thiamine, and light (Lys0) l-lysine were purchased from Merck (Burlington, MA). Na_2_HPO_4_·7H_2_O and KH_2_PO_4_ were purchased from Thermo Fisher Scientific (Waltham, MA) and Avantor (Radnor, PA). Medium (Lys6) and heavy (Lys8) isotopes were purchased from Cambridge Isotope Laboratories (Tewksbury, MA). E. coli K-12 auxotrophic for lysine (strain JW2806-1, from the Keio collection of single gene knockouts) was employed in the experiments conducted in this study.

### Bacterial cell culture and pulsed-SILAC labeling.

E. coli cultures were grown over night on M9 medium (5× M9 salts [0.24 M Na_2_HPO_4_· 7H_2_O, 0.11 M KH_2_PO_4_, 42.8 mM NaCl, 93.45 mM NH_4_Cl], 2 mM MgSO_4_, 0.4% glucose, 0.1 mM CaCl_2_, 0.1 mg/ml of thiamine) agar plates supplemented with 250 μg/ml of lysine and 50 μg/ml of kanamycin. For isotope labeling, two single colonies were passaged twice at 37°C on M9 medium containing 250 μg/ml of SILAC residues, either light (L), or medium (M). Samples from the two cultures were then reseeded at a low optical density (OD) (OD_M_ at 600 nm = 0.033; OD_L_ at 600 nm = 0.03) in fresh M or L M9 medium. Upon early log phase (OD_M_ at 600 nm = 0.343; OD_L_ at 600 nm = 0.267), two samples were taken: one of M labeled cells that was used for verification of full incorporation of the M lysine isotope (>98% incorporation) and one that was a mixture of equivalent amounts of cells from the L and M cultures (*t*_0 h_). At that time point, the M-containing culture medium was replaced with an equivalent volume of heavy (H)-containing medium (250 μg/ml), while the L-containing culture medium was replaced with an equivalent volume of fresh L-containing medium, using rapid filtration on 0.22-μm filters. Following medium exchange, the culture now growing in H medium was sampled at five time points (*t*_0.25 h_, *t*_1 h_, *t*_2 h_, *t*_3 h_, and *t*_4 h_) and mixed with an equivalent amount of cells growing in the L medium. The cells were harvested by centrifugation at 4,000 × *g* and 4°C for 10 min, resuspended in 1 ml of M9 medium, snap-frozen in liquid nitrogen, and stored at −80°C. The experimental setup is illustrated in Fig. 1.

### Proteomics.

Sample preparation, liquid chromatography, mass spectrometry, and data processing were done at the De Botton Protein Profiling Institute of the Nancy and Stephen Grand Israel National Center for Personalized Medicine, Weizmann Institute of Science.

### Sample preparation.

All chemicals were purchased from Sigma-Aldrich unless otherwise noted. Cell pellets were lysed with 5% SDS in 50 mM Tris-HCl. Lysates were incubated at 96°C for 5 min, followed by six cycles of 30 s of sonication (Bioruptor Pico; Diagenode, USA). Protein concentration was measured using the bicinchoninic acid (BCA) assay (Thermo Scientific, USA), and a total of 30 μg protein was reduced with 5 mM dithiothreitol and alkylated with 10 mM iodoacetamide in the dark. Each sample was loaded onto S-Trap microcolumns (Protifi, USA) according to the manufacturer’s instructions. In brief, after loading, samples were washed with 90%:10% methanol/50 mM ammonium bicarbonate and digested with LysC (1:50 protease/protein) for 1.5 h at 47°C. The digested peptides were eluted with 50 mM ammonium bicarbonate and incubated overnight with trypsin at 37°C. Two additional elutions were performed using 0.2% formic acid and 0.2% formic acid in 50% acetonitrile. The three elutions were pooled and vacuum centrifuged to dry. Samples were kept at −80°C until analysis.

### Liquid chromatography.

LC/MS-grade solvents were used for all the chromatographic steps. Each sample was loaded using splitless nano-ultraperformance liquid chromatography (nano-UPLC) (10,000-lb/in^2^ nanoAcquity; Waters, Milford, MA). The mobile phases were H_2_O plus 0.1% formic acid (mobile phase A) and acetonitrile plus 0.1% formic acid (mobile phase B). Desalting of the samples was performed online using a reversed-phase Symmetry C_18_ trapping column (180-μm internal diameter, 20-mm length, and 5-μm particle size; Waters). The peptides were then separated on a T3 high-strength silica nanocolumn (75-μm internal diameter, 250-mm length, and 1.8-μm particle size; Waters) at 0.35 μl/min. Peptides were eluted from the column into the mass spectrometer using the following gradient: 4% to 25% buffer B in 155 min, 25% to 90% buffer B in 5 min, maintenance at 90% for 5 min, and then back to initial conditions.

### Mass spectrometry.

The nano-UPLC was coupled online through a nano-electrospray ionization (nano-ESI) emitter (10-μm tip; New Objective; Woburn, MA) to a quadrupole Orbitrap mass spectrometer (Q Exactive HF; Thermo Scientific) using a FlexIon nanospray apparatus (Proxeon). Data were acquired in data-dependent acquisition (DDA) mode, using a Top20 method. MS1 resolution was set to 120,000 (at 400 *m/z*), mass range of 375 to 1,650 *m/z*, automatic gain control of 3E6, and maximum injection time was set to 60 ms. MS2 resolution was set to 15,000, quadrupole isolation 1.7 *m/z*, automatic gain control (AGC) of 1e5, dynamic exclusion of 45 s, and maximum injection time of 60 msec.

### Data processing.

Raw data were processed with MaxQuant version 1.6.0.16 (66). The data were searched with the Andromeda search engine (67) against the UniProt E. coli K-12 proteome database (UP000000625) appended with common lab protein contaminants and the following modifications: Carbamidomethylation of C as a fixed modification and oxidation of M and deamidation of N and Q as variable ones. Labeling was defined as follows: H, heavy K8; M, medium K4; and L, light K0. The match between runs option was enabled as well as the requantify function. The rest of the parameters were used as default. Decoy hits were filtered out using Perseus version 1.6.0.7 (68), as well as proteins that were identified on the basis of a modified peptide only.

### Determination of protein half-life.

We used a modeling scheme similar to that described in reference 28. As stated above, we sampled bacteria from two different cultures, grown in either L- or M-containing medium. At time zero, the M-containing medium was replaced with H-containing medium. Let $\hat{L}$ be the abundance of the L isotope in cells grown in L-containing medium (i.e., the number of protein molecules harboring the L isotope). We assume that in each generation, the number of cells is doubled and consequently, $\hat{L}$ is doubled as well. Let *t*_cc_ be the generation time in minutes (∼60 min in our cultures). Thus, when the cells are growing for *t* minutes, the number of generations is *t*/*t_cc_* and the total abundance of the integrated L isotope is:

(1) $$\hat{L}=L_{0}2^{\frac{t}{t_{\text{cc}}}}$$

Let $\hat{M}$ be the abundance of the M isotope in cells grown in M-containing medium. Following removal of the M-containing medium at time zero, $\hat{M}$ is expected to have an exponential decay with a specific rate factor. We note that cell division does not affect $\hat{M}$, because $\hat{M}$ measures the total amount of M in the cells. Thus, $\hat{M}$ is expected to decrease due to protein degradation according to the following equation:

(2) $$\hat{M}=M_{0}e^{-t\lambda_{\text{deg}}}$$

The parameter $\lambda_{\text{deg}}$ governs the degradation rate. High values of $\lambda_{\text{deg}}$ indicate higher rates of degradation, and at the limit, when $\lambda_{\text{deg}}$ *=* 0, the abundance $\hat{M}$ remains *M*_0_ regardless of *t*.

Up until the medium replacement step, $\hat{M}=\hat{L}$ (because these two isotopes are used in parallel under the same conditions). Upon medium replacement, the M isotope available in the medium is washed away by filtration and replaced with H isotope in medium. We do the same procedure for the L isotope: the L medium is washed away and replaced with fresh L-containing medium (Fig. 1). Thus, at the replacement time point,$\;\hat{L}=\hat{M}$, and after this time point, added L in the cells is the same as added H in the cells. Thus, the total abundance of L in the cells should equal the sum of the integrated M and H isotopes:

(3) $$\hat{L}=\hat{M}\;+\;\hat{H}$$

Taking the next samples, at each time point, we made sure to take the same number of cells from the L culture and from the culture of H plus M. Hence, the measured levels of L and M at time point *t* are:

(4) $$L\left(t\right)=L_{0}2^{\frac{t}{t_{\text{cc}}}}f\left(t\right)$$

(5) $$M(t)=M_{0}e^{-t\lambda_{\text{deg}}}f(t)$$

where f(t) is the fraction of cells sampled at time *t*. From these equations, we obtain

(6) $$\frac{M(t)}{L(t)}=\frac{M_{0}}{L_{0}}\frac{e^{-t\lambda_{\text{deg}}}}{e^{\frac{t}{t_{\text{cc}}}ln2}}=\frac{M_{0}}{L_{0}}e^{-t(\lambda_{\text{deg}}+\frac{\ln2}{t_{\text{cc}}})}$$

In our experiments, the proteomic results after MaxQuant analysis provide us with the $\frac{M(t)}{L(t)}$ and $\frac{H(t)}{L(t)}$ observed values. In theory, according to equation 3, these two ratios should sum up to 1. In practice, however, small deviations from the sum of 1 are observed (0.99 ± 0.01, at 95% confidence interval). Hence, we add a normalization step in which we multiply both ratios by a fixed constant so that they sum to 1 for every *t*. Also note that according to the experimental design, *M*_0_ should equal *L*_0_, and thus, their ratio should be 1. In our experiment, we observed a ratio of 1.02 ± 0.02, at 95% confidence interval. The normalized $\frac{M(t)}{L(t)}$ ratios are plotted against *t*, where M(t) and L(t) represent the observed intensity of the medium and light isotopes at each time point, respectively. Using R’s nonlinear least-squares routine, *nls* (69), we then fit the obtained curve to a simple exponential function of the form

(7) $$y\left(t\right)=Ae^{-t(\lambda_{\text{dil}}+\lambda_{\text{deg}})}\;+\;B$$

The estimated parameters in this nonlinear regression are A, B and $\lambda_{\text{deg}}$. Comparing equations 6 and 7, A corresponds to the normalized $\frac{M(t)}{L(t)}$ ratio at $t=0,\lambda_{\text{deg}}$ corresponds to the degradation constant, and B accounts for the offset seen in data, which is attributed to recycling in reference 28. $\lambda_{\text{dil}}=\frac{\ln\;2\;}{t_{\text{cc}}}$ is the dilution constant, where $t_{\text{cc}}=60\text{ min}$.

Proteins that obey the following criteria were omitted from the data set before fitting the model:

• Less than four measurements.
• Proteins that cannot be distinguished based on the respective peptides identified by MS.
• Proteins that were identified using less than two peptides.

### Likelihood ratio test.

Early studies have shown that during exponential growth under standard conditions, the E. coli proteome is stable, suggesting that for the vast majority of E. coli’s proteome under these conditions, the degradation constant is practically zero (47, 48, 58, 70–72). We therefore formulated two nested protein degradation models based on equation 7. The first model states that for a given protein, $\lambda_{\text{deg}}=0$:

(8) $$y\left(t\right)=Ae^{-t\lambda_{\text{dil}}}\;+\;B$$

whereas the second model states that $\lambda_{\text{deg}}$ is a free parameter, $\lambda_{\text{deg}}\;>\;0$:

(9) $$y\left(t\right)=Ae^{-t(\lambda_{\text{dil}}+\lambda_{\text{deg}})}\;+\;B$$

R’s *nls* was used to estimate the parameters fit, using the nl2sol algorithm from the Port library (73). The *nl2sol* algorithm allows setting boundaries for the estimated parameters. For both models, A and B were limited to [0.75, 1.25] and [0, 0.4], respectively, while for the second model, $\lambda_{\text{deg}}$ was limited to [0, $100\times \lambda_{\text{dil}}$]. These boundaries enabled omitting proteins for which the offset, *B*, is higher than the initial isotopic ratio, *A*, as well as to prevent $\lambda_{\text{deg}}$ from being estimated negative, which is biologically impossible. We constrained $\lambda_{\text{deg}}$ to be at most 100-fold more effective than $\lambda_{\text{dil}}$, to prevent the estimation of half-life (see below) to near 0, which is also impossible. Using R’s *lrtest* function, the likelihood ratio test was then employed to select the model that best fits the data. The *P* values returned by the *lrtest* function were then corrected for multiple testing using the Benjamini-Hochberg correction, using R’s *p.adjust*. Proteins for which the *P* value was equal to, or larger than, 0.05 were labeled as stable, whereas the rest of the proteins were labeled as degradable. In the case of the degradable group, the fitted $\lambda_{\text{deg}}$ was used to calculate the half-life, *t*_1/2_:

(10) $$t_{1/2}=\frac{\ln\;2}{\lambda_{\text{deg}}}$$

Proteins with fits of low quality (*R*^2^ < 0.8) in both models were discarded. No statistically significant functional enrichment/depletion was detected among these proteins after adjusting the *P* values according to the Benjamini-Hochberg procedure (data not shown).

### Expectation maximization algorithm.

To determine which degradable proteins are fast or slow degrading, we used the *mixR* package for expectation maximization. The calculated *t*_1/2_ was given as an input to *mixR*’s function, *mixfit*, which performs maximum likelihood estimation for various finite mixtures using the expectation maximization algorithm. The statistical significance of the mixture model was estimated using *mixR*’s *bs.test* function. A probability threshold of 0.5 was used to attribute each observation to the respective component. Setting the probability threshold to values higher than 0.5 had an insubstantial effect on downstream analyses (data not shown).

### Enrichment analysis.

Gene ontology (GO) molecular function and biological pathways and Kyoto Encyclopedia of Genes and Genomes (KEGG) pathway annotations were analyzed for enrichment using Perseus (68).

### Feature extraction.

A total of 188 structural, physical, protein-protein interaction network (PPIN), and physicochemical features were collected (Data Set S2). Four features describing the intrinsic disorder propensity were extracted using the ESpritz 1.3v webserver (74): (i) fraction of disordered amino acids out of the total protein length, (ii) total number of disordered segments, (iii) total number of disordered segments composed of at least 30 amino acids, and (iv) total number of disordered segments composed of at least 50 amino acids. Six additional features describing the PPIN of a protein were extracted from the STRING 11.0v database (75): (i) the total number of interacting partners of a protein by counting all its neighbors (node connectivity), (ii) the average pI of interacting partners, (iii) the average molecular weight of interacting partners, (iv) the average sequence length of interacting partners, (v) the average disorder among interacting partners (calculated by dividing the total number of disordered amino acids across all interacting partners by the total length of interacting partners), and (vi) a binary feature describing whether a protein is an isolated node (i.e., a node without neighbors) in the PPIN. An additional 128 PPIN features were extracted using node2vec (76). These features are extracted for each node in the network: in our case, each node is a protein, and the network is the network of protein-protein interaction. Isolated nodes were assigned with zeroes. The PPIN was predicted by STRING using only those proteins that were detectable at at least three time points (1,223 proteins). The node2vec algorithm encodes each node as a point in a high-dimension space (by default, 128 dimensions). Each coordinate is considered a feature, and thus, each node is characterized by 128 features. The encoding aims to place nodes that share similar neighborhood properties close to each other in the high-dimensional space. More formally, neighborhood similarity is defined based on random walks starting from each node. Notably, the features are not *a priori* defined; rather, they are inferred as part of the node2vec algorithm. Unfortunately, the biological interpretation of the node2vec features is unclear. Ten additional features were extracted using the ProteinAnalysis class of the Biopython package (77): (i) molecular weight, (ii) average protein aromaticity (78), (iii) average protein instability (79), (iv) isoelectric point, (v) average gravy score (80), (vi) average flexibility (81), (vii) sequence length, (viii) fraction of helix positions, (ix) fraction of turn positions, and (x) fraction of beta sheet positions. Ten additional features were calculated by dividing each of the Biopython features by the number of interacting partners of each protein. To handle isolated nodes (four proteins), we artificially added one neighbor to all proteins in the network. Twenty additional features consist of the number of occurrences of each of the 20 amino acids at the second position of the N terminus. Five additional features consist of the number of occurrences of each of the 20 amino acids grouped into five physicochemical groups at the second position of the N terminus: (i) aliphatic (IVL), (ii) aromatic (FYWH), (iii) charged (KRDE), (iv) tiny (GACS), and (v) diverse (TMQNP). Five additional features consist of the number of occurrences of five different previously described degradation signals: three N-terminal signals termed NM1 (polar-T/ϕ-ϕ-basic-ϕ), NM2 (NH_2_-Met-basic-ϕ-ϕ-ϕ-X5-ϕ), and NM3 (ϕ-X-polar-X-polar-X-basic-polar) and two C-terminal signals termed CM1 (LAA-COOH) and CM2 (RRKKAI-COOH).

### Comparative analysis of protein features.

One-way analysis of variance (ANOVA) followed by Tukey’s test was used to test for statistical significance in isoelectric point, mass, percent disorder, and number of interacting partners in the PPIN among the three stability groups. Chi-square test was used to analyze differences among groups for binary features, e.g., presence/absence of a sequence-related motif. All *P* values are reported after a Benjamini-Hochberg false-discovery rate (FDR) correction.

### Machine learning protocol.

Classification between several grouping of the proteins was tested: (i) fast- versus slow-degrading proteins, (ii) fast-degrading versus stable proteins, (iii) slow-degrading versus stable proteins, (iv) fast-degrading proteins versus the rest of the proteins; (v) slow-degrading proteins versus the rest of the proteins, (vi) stable versus the rest of the proteins, and (vii) fast-degrading proteins versus slow-degrading proteins versus stable proteins. We aimed to test whether machine learning can be used to classify the open reading frames (ORFs) into distinct stability groups. We used least absolute shrinkage and selection operator (LASSO) regularized logistic regression (82) for each classification task for its speed, robustness, and interpretability. Model training was performed via the Python package scikit-learn (83) using the optimization algorithm liblinear. The penalty parameter for regularization was determined by nested cross-validation. All learning was based on the 1,149 ORFs for which we could determine protein degradation rates (see Results).

The performance of the classification was measured in terms of AUC. The performance on the actual data was estimated by 10 repetitions of 10-fold cross-validation; i.e., 90% of the data were randomly chosen for training the model, and the remaining 10% were used for testing the performance of the classification. This was done in a stratified manner, i.e., keeping the relative frequency of the two groups the same in each fold. In each repetition, 10-fold cross-validation is repeated with different randomization of the split to train and test sets. The AUC of each 10-fold cross-validation was calculated by averaging the AUC over the 10 folds. For classification of the three stability groups, the same approach was taken except that scikit-learn’s multinomial logistic regression was used, and the performance of the classification was measured in terms of one-versus-rest AUC, in which the AUC of each class was calculated against the rest. The reported AUC is the average over the three one-versus-rest AUCs.

To test whether the AUC is significantly higher than random, class labels (stable/slow degrading/fast degrading) were randomly shuffled among all proteins. The same inference as described above was conducted on the permuted data. This was repeated 100 times. One-way ANOVA followed by Tukey’s test was used to compare the performance of the classifier on the actual versus permuted data.

We tried alternative machine learning classifications (random forest, K nearest neighbors, SVM with various kernels, linear discriminate analysis, and naive Bayes, with and without dimensionality reduction using principal-component analysis), which did not provide any significant increase in classification accuracy (data not shown). In addition, we considered including various features such as all pairs of amino acids (400 features) and all triplets (8,000 features). Their inclusion did not contribute to classification accuracy and the data are hence not shown.

### Data availability.

The mass spectrometry proteomics data have been deposited to the ProteomeXchange Consortium via the PRIDE (84) partner repository with the data set identifier PXD022112.
