# Supplementary material for: Harnessing Machine Learning To Unravel Protein Degradation in Escherichia coli
Source: mSystems. 2021 Feb 2;6(1):e01296-20. doi: 10.1128/mSystems.01296-20 (PMC7857536; doi:10.1128/mSystems.01296-20)
Supplement: TABLE S1 [file mSystems.01296-20-st001.pdf]

Gene	Protease	Ref	Measured Half-life
rpoS	ClpXP	(1)	6
leuD	ClpAP	(2)	66
fliC	Lon	(3)	7
ybjX	FtsH	(4)	17
lpxC	FtsH	(4)	60
proV	Lon	(5)	35
lipA	Lon, ClpXP	(5)	29
sodC		(6)	62
uvrY	ClpAP	(7)	45
bioB		(8)	13
clpA	ClpAP	(9)	14
relB	Lon	(10)	32
dinJ	Lon, ClpXP	(10)	20
iscU	ClpXP	(11)	15
dps	ClpXP, ClpAP	(11)	6
mqsA	Lon	(11)	9
ydaM	ClpXP	(12)	14
katE	ClpXP	(12)	26
pepB	ClpXP	(12)	50
acnB	ClpXP	(12)	53

cysD	ClpXP	(12)	59
murD			1
macB			1
slyA			6
sbp			7
patA			7
cspB			9
sodB			12
iscA			13
cspA			13
yciA			14
cfa			14
ycgB			15
cysH			15
clpS			16
tatE			16
codA			19
yoaC			22
intF			23
pyrI			25
nagZ			29
sra			32

acnA	32
deaD	33
miaB	35
rpsO	36
proX	39
erpA	40
ydhQ	40
argF	42
raiA	43
holE	44
ygiB	46
ydjY	46
cysC	47
mrr	48
yeaH	49
alsB	51
yjdJ	53
rfaC	54
oppD	56
ttcA	61
ggt	65
phnA	66

mgtA	66
elaA	66
nuoG	67
fumC	67
proP	68
amiA	70
speA	70
pspE	70

Bibliography

1. Becker G, Klauck E, Hengge-Aronis R. 1999. Regulation of RpoS proteolysis in *Escherichia coli*: The response regulator RssB is a recognition factor that interacts with the turnover element in RpoS. *Proc Natl Acad Sci U S A* **96**:6439–6444.

2. Weichart D, Querfurth N, Dreger M, Hengge-Aronis R. 2003. Global Role for ClpP-Containing Proteases in Stationary-Phase Adaptation of *Escherichia coli*. *J Bacteriol* **185**:115–125.

3. Lu Y, Welsh JP, Chan W, Swartz JR. 2013. *Escherichia coli*-based cell free production of flagellin and ordered flagellin display on virus-like particles. *Biotechnol Bioeng* **110**:2073–2085.

4. Arends J, Thomanek N, Kuhlmann K, Marcus K, Narberhaus F. 2016. In vivo trapping of FtsH substrates by label-free quantitative proteomics. *Proteomics* **16**:3161–3172.

5. Arends J, Griego M, Thomanek N, Lindemann C, Kutscher B, Meyer HE, Narberhaus F. 2018. An Integrated Proteomic Approach Uncovers Novel Substrates and Functions of the Lon Protease in *Escherichia coli*. *Proteomics* **18**:1800080.

6. Sakurai Y, Anzai I, Furukawa Y. 2014. A primary role for disulfide formation in the productive folding of prokaryotic Cu,,Zn-superoxide dismutase. *J Biol Chem* **289**:20139–20149.

7. Yeom J, Gao X, Groisman EA. 2018. Reduction in adaptor amounts establishes degradation hierarchy among protease substrates. *Proc Natl Acad Sci U S A* **115**:E4483–E4492.

8. Reyda MR, Dippold R, Dotson ME, Jarrett JT. 2008. Loss of iron-sulfur clusters from biotin synthase as a result of catalysis promotes unfolding and degradation. *Arch Biochem Biophys* **471**:32–41.

9. Maglica Ž, Striebel F, Weber-Ban E. 2008. An Intrinsic Degradation Tag on the ClpA C-Terminus Regulates the Balance of ClpAP Complexes with Different Substrate Specificity. *J Mol Biol* **384**:503–511.

10. Brzozowska I, Zielenkiewicz U. 2013. Regulation of toxin-antitoxin systems by proteolysis. *Plasmid* **70**:33–41.

11. Pruteanu M, Baker TA. 2009. Proteolysis in the SOS response and metal homeostasis in Escherichia coli. *Res Microbiol* **160**:677–683.

12. Flynn JM, Neher SB, Kim YI, Sauer RT, Baker TA. 2003. Proteomic discovery of cellular substrates of the ClpXP protease reveals five classes of ClpX-recognition signals. *Mol Cell* **11**:671–683.
