# Supplementary figures and images for: Harnessing Machine Learning To Unravel Protein Degradation in Escherichia coli
Source: mSystems. 2021 Feb 2;6(1):e01296-20. doi: 10.1128/mSystems.01296-20 (PMC7857536; doi:10.1128/mSystems.01296-20)

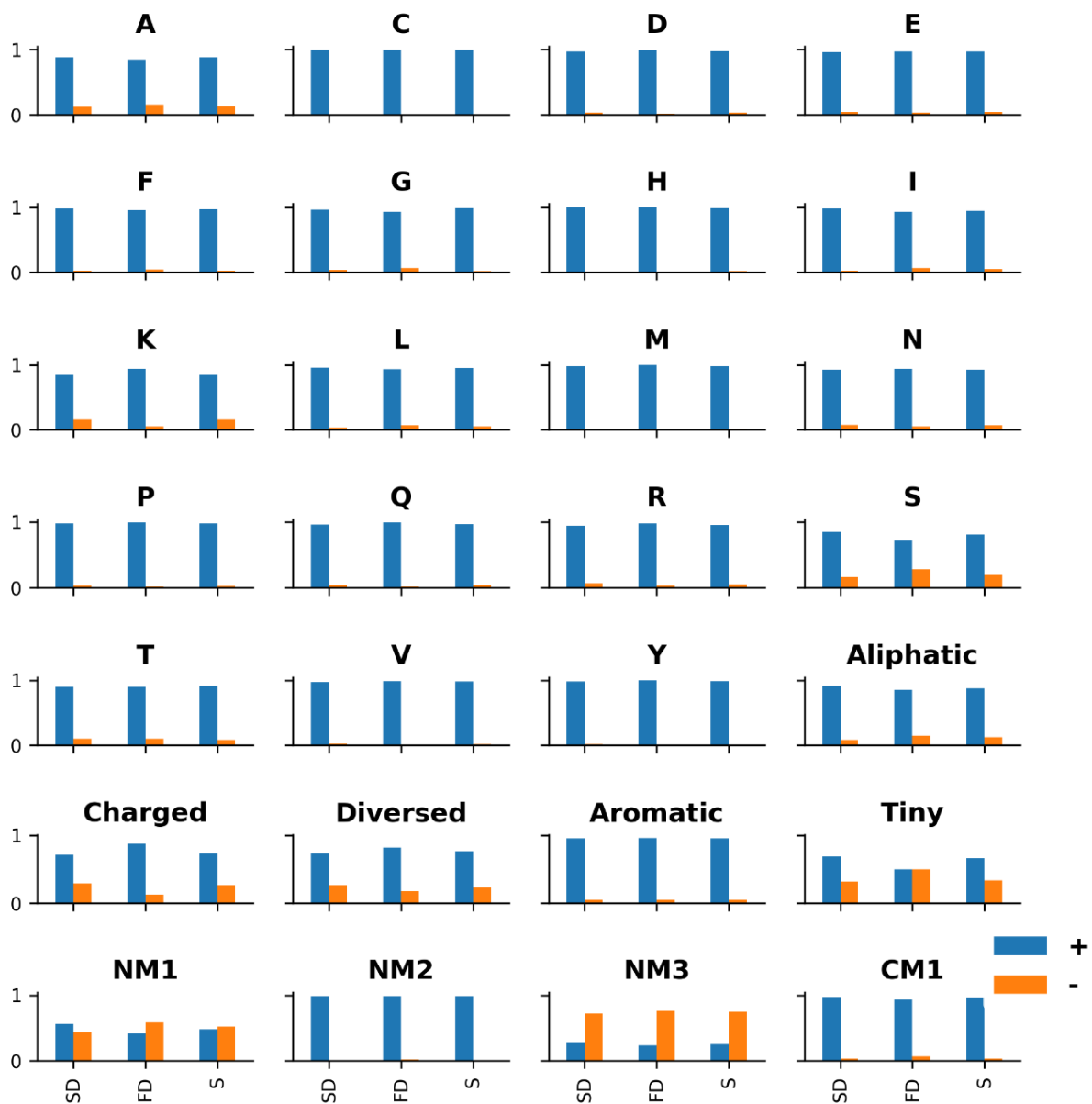

Supplement: FIG S1 [file mSystems.01296-20-sf001.pdf]

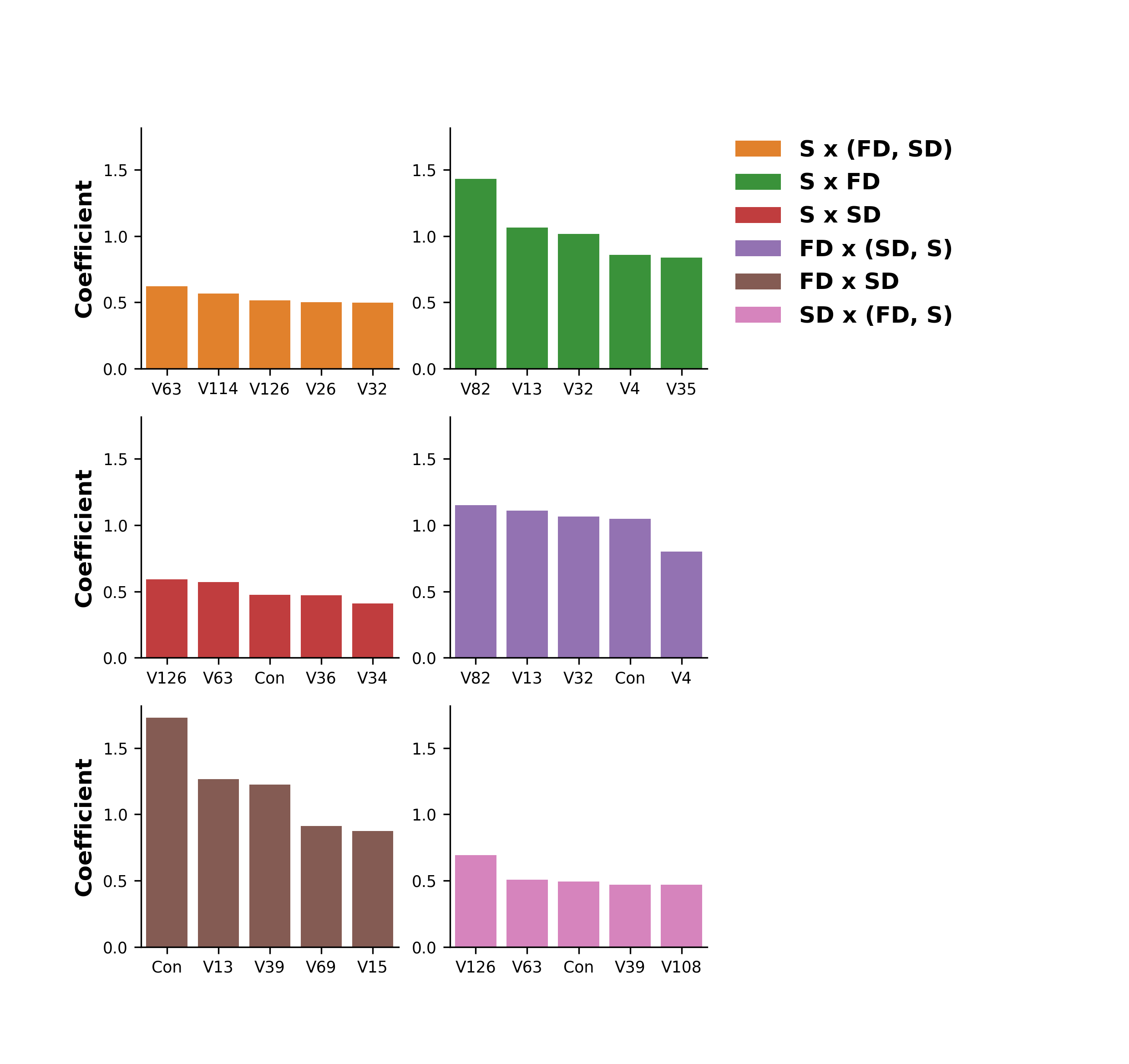

Supplement: FIG S2 [file mSystems.01296-20-sf002.tif]
